# The Clinical and Endoscopic Profiles of Patients With Upper Gastrointestinal Bleeding (UGIB) and the Role of the Rockall Scoring System in Predicting Adverse Outcomes

**DOI:** 10.7759/cureus.40418

**Published:** 2023-06-14

**Authors:** Anshul, Navpreet Singh, Hardik Pahuja, Vineet Kumar, Bhuvan Priyanshu Popli, Sachin Kumar

**Affiliations:** 1 Anesthesiology, Pandit Bhagwat Dayal Sharma Post Graduate Institute of Medical Sciences, Rohtak, IND; 2 Internal Medicine, Gian Sagar Hospital, Rajpura, IND; 3 Medicine, Gian Sagar Hospital and Medical College, Rajpura, IND; 4 Internal Medicine, Dayanand Medical College and Hospital, Ludhiana, IND; 5 Internal Medicine, Maulana Azad Medical College, Delhi, IND; 6 Anesthesiology, All India Institute of Medical Sciences, New Delhi, Delhi, IND

**Keywords:** rockall scoring system, chronic liver disease, upper gastrointestinal tract, esophageal varices, endoscopy

## Abstract

Introduction: Upper gastrointestinal bleeding (UGIB) is one of the common emergencies seen by physicians. Upper gastrointestinal (UGI) endoscopy remains a crucial tool in the identification of UGIB.

Objective: The aim of the present study was to determine the clinical and endoscopic profiles of UGIB in an adult population.

Methods: This prospective, cross-sectional study was conducted in Dayanand Medical College and Hospital (DMCH), Ludhiana, where 75 patients aged 18 years and above admitted to the hospital with a history of UGIB from July 1 to December 31, 2018, were enrolled in the study. After obtaining the demographic data, all patients underwent clinical examination, laboratory investigations, and video endoscopy. The Rockall scoring system was used to assess their prognosis.

Results: The mean age of the study population was 52.19±6.65 years. The majority (33%) were in the age group of 51-60 years. Of the study population, 82.7% were male and 17.3% were female. Chronic alcohol intake was found to be the most common risk factor, followed by drug intake. On upper gastrointestinal endoscopy, esophageal varices (65.3%) were the most common finding, followed by peptic ulcer disease (25.2%), gastric erosions (2.6%), gastroduodenitis (1.3%), Mallory-Weiss tear (1.3%), carcinoma stomach (1.3%), Camron’s lesion (1.3%), and Dieulafoy’s lesion (1.3%). Mortality attributed to UGIB was found to be 8%.

Conclusion: The present study reported portal hypertension as the most common cause of UGIB, while the most common endoscopic lesions reported were esophageal varices. The factors associated with poor prognosis were age >60 years, shock, respiratory failure, low hemoglobin, low platelet count, deranged international normalized ratio (INR), variceal bleed, renal failure, rebleed, Rockall score ≥ 8, and late endoscopy (>24 hours of admission). Urgent appropriate hospital management definitely helps to reduce morbidity and mortality in patients with UGIB.

## Introduction

Upper gastrointestinal bleeding (UGIB) refers to blood loss within the intraluminal gastrointestinal (GI) tract from any location between the upper esophagus to the duodenum proximal to the ligament of Treitz [1]. The most common presenting features of UGIB are hematemesis, melena, and shock. To produce clinical signs, the patient should bleed at least 500 mL of blood within three hours [2,3].

Patients can have either variceal or non-variceal bleeding. Both variceal and non-variceal bleeding have different treatment algorithms and prognoses [4]. The causes of UGIB vary by region, lifestyle, and healthcare hierarchy. Portal hypertension, peptic ulcer bleeding, arteriovenous malformations, Mallory-Weiss tear, gastritis and duodenitis, and malignancy are various etiological factors of UGIB [2].

The primary diagnostic test for the evaluation of UGIB is endoscopy [5]. The ability to take targeted mucosal biopsies remains a unique strength of endoscopy as compared to other radiological imaging studies [6]. Early endoscopy helps in the early diagnosis of lesions, thus reducing rebleed, the requirement for transfusion, the need for surgery, costs, and the duration of hospitalization [5].

The Rockall scoring system identifies patients at higher risk of rebleed and mortality [5]. It is a risk assessment score to predict clinically relevant outcomes, including mortality, the need for hospital-based intervention, rebleeding, and length of hospital stay [7].

Most of the studies and trials have been carried out to look for the incidence of various etiologies, risk assessment for rebleed and adverse outcomes, the role of early and late endoscopy, and restrictive blood transfusion, but there is a paucity of data on the clinical and endoscopic profile of patients with UGIB [2]. Therefore, this study was planned with the primary objective of identifying the clinical and endoscopic profiles of patients presenting with UGIB in the emergency department of Dayanand Medical College and Hospital (DMCH). The secondary objective was to check the positive predictive value of the Rockall scoring system in the identification of patients at risk of adverse outcomes.

## Materials and methods

This observational, cross-sectional study was conducted in the Department of Medicine in collaboration with the Department of Gastroenterology at DMCH, Ludhiana, after approval by the Institutional Ethics Committee vide letter number DMCH/4/15-2018 and after obtaining informed written consent from patients. Eighty-nine patients (age more than 18 years) presented with a chief complaint of UGIB, i.e., hematemesis and/or melena, over the duration of six months, i.e., from July 1 to December 31, 2018, in the emergency department of our hospital. Thirteen patients refused upper gastrointestinal (UGI) endoscopy, and one was lost to follow-up. The rest of the 75 patients were enrolled in the study.

The clinical and endoscopic profiles of all patients were assessed according to pretested and predesigned proforma. Data regarding demographic details, chief complaints, risk factors, and comorbidities for UGIB (alcoholism, nonsteroidal anti-inflammatory drugs, antiplatelets, smoking, diabetes, hypertension, and coronary artery disease) were documented. Clinical history, examination including presenting symptoms of the patients, day of hospitalization after bleeding along with a previous history of hematemesis, melena, hematochezia, or syncope, and vital parameters such as pulse rate, systolic and diastolic blood pressures, and respiratory rate were recorded.

Complete hemogram (CH), stool routine, microscopy and occult blood, urine routine, liver function tests (LFTs), prothrombin time-international normalized ratio (PT-INR), activated partial thromboplastin clotting time (aPTT), renal function tests (RFTs), RBC indices, random blood sugar (RBS), and USG of the abdomen were carried out in all patients. The Rockall score for each patient was calculated and used to predict the prognosis and chances of rebleeding. A score of <3 carries a good prognosis, but a total score of >8 carries a high risk of mortality [8]. Table 1 shows various components of the Rockall scoring system [5].

**Table 1:** Distribution of patients according to chief complaints SBP: systolic blood pressure, HR: heart rate, IHD: ischemic heart disease, GI: gastrointestinal

Age	
<60 years	0
60-79 years	1
>80 years	2
Shock	
No shock, SBP > 100 mmHg, HR < 100/minute	0
Tachycardia, SBP > 100 mmHg, HR > 100/minute	1
Shock, SBP < 100 mmHg	2
Comorbidities	
No major comorbidity	0
Cardiac failure, IHD, or any other major comorbidity	2
Renal failure, liver failure, or any metastatic disease	3
Diagnosis	
Mallory-Weiss tear, no lesion seen, no stigmata of recent bleed	0
All other diagnoses apart from GI malignancy	1
GI malignancy	2
Major stigmata of recent bleed	
None or dark spots only	0
Blood, adherent clot, spurting vessel	2

Acute bleeding was managed with a multifactorial approach, including blood transfusion and the use of vasoactive drugs. Once the patient was stable hemodynamically after initial resuscitation, UGI endoscopy was performed using an Olympus videoscope (Olympus, Tokyo, Japan), and the following parameters were recorded: timing of endoscopy, whether the patient has undergone any intervention, i.e., banding/clipping/sclerotherapy during this procedure, and record of rebleed including the day of rebleed after the initial endoscopy. Rebleed was assessed until one month before the follow-up period. The outcome was assessed in terms of discharge in stable condition, discharge against medical advice (DAMA), and inhospital or 30-day mortality.

Statistical analysis

Data were described in terms of range, mean±standard deviation (±SD), median, frequencies (number of cases), and relative frequencies (percentages) as appropriate. A comparison of quantitative variables was made using paired t-test for parametric data. For comparing categorical data, the Chi-square (χ2) test was performed, and the exact test was used when the expected frequency was less than 5. A probability value (p-value) of less than 0.05 was considered statistically significant. All statistical calculations were done using Statistical Package for the Social Sciences (SPSS) version 21 for Microsoft Windows (IBM SPSS Statistics, Armonk, NY, USA).

## Results

**Figure 1:**
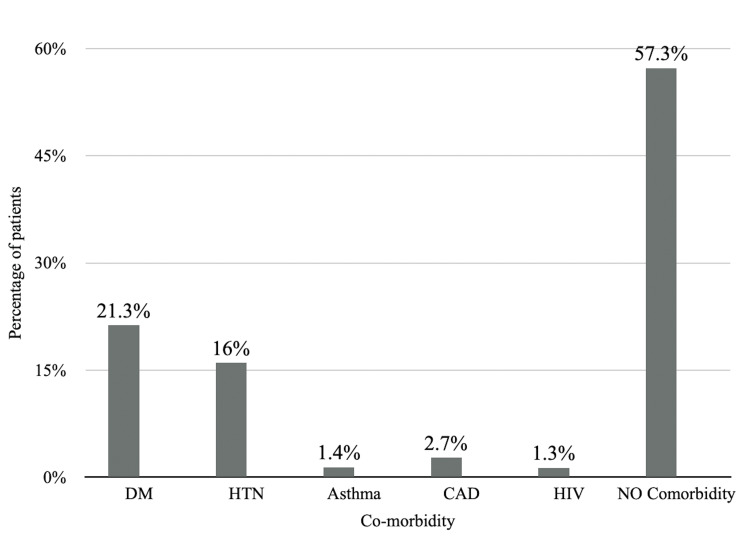
Distribution of patients according to the presence of comorbidities DM: diabetes mellitus, HTN: hypertension, CAD: coronary artery disease, HIV: human immunodeficiency virus

Out of 75 subjects, 62 were males and 13 were females. The mean age of the study population was 52.19±6.65 years, and the majority (33.3%) were in the age group of 51-60 years. Figure 1 shows the distribution of patients according to the presence of comorbidities.

At the time of presentation, 43.9% of the patients had hemoglobin (Hb) below 7 g/dL. The mean Hb of patients who presented with hematemesis was 7.5±2.9 g/dL, those who presented with melena was 5.6±1.6 g/dL, and those who presented with both hematemesis and melena was 7.4±2.8 g/dL. Of the patients, 81.3% had platelet counts of less than 150 × 10^9^/L of blood, and 76% of patients had deranged INR, i.e., more than 1.5. Serum albumin of <3.5 g/dL was found in 59.7% of patients. Table 2 shows the distribution of patients according to endoscopic findings.

**Table 2:** Distribution of patients according to endoscopic findings EV: esophageal varices, CA stomach: carcinoma of the stomach, GEJ ulcers: gastroesophageal ulcers

Endoscopic finding		Number of patients	Percentage
EV		49	65.3%
Non-variceal causes	Esophagitis + ulcer	4	5.2%
	Esophageal + gastric ulcer	2	2.6%
	Gastric ulcers	3	4%
	Gastric + duodenal ulcer	2	2.6%
	Duodenal ulcers	6	8%
	Gastric erosions	2	2.6%
	GEJ ulcers	2	2.6%
	Gastroduodenitis	1	1.3%
	Mallory-Weiss tear	1	1.3%
	CA stomach	1	1.3%
	Cameron’s lesion	1	1.3%
	Dieulafoy’s lesion	1	1.3%

**Figure 2:**
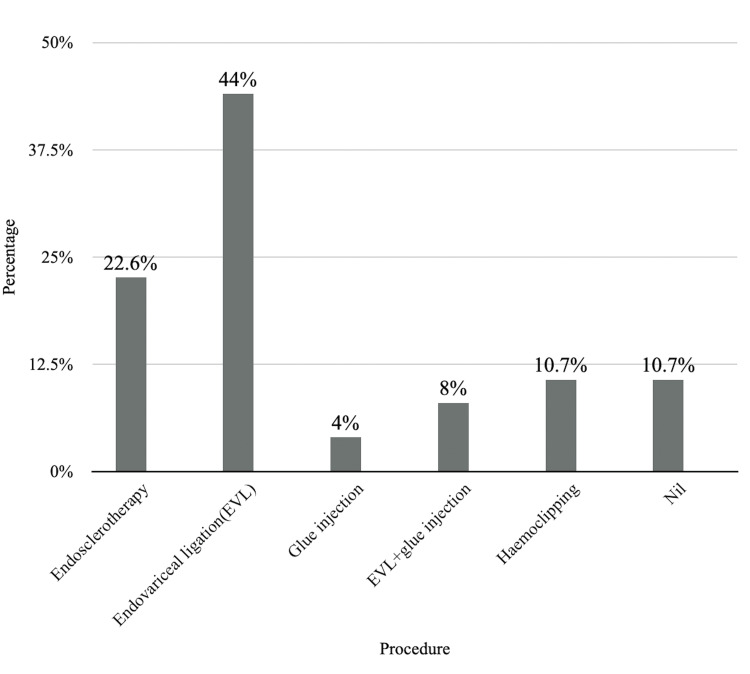
Distribution of patients according to the procedure done during endoscopy EVL: endovariceal ligation

Figure 2 shows the distribution of patients according to the procedure done during endoscopy.

Twelve (16%) patients rebled during hospital stays after the endoscopic treatment. Among 75 patients, 64 (85%) were discharged in stable condition, six (8%) had inhospital or 30-day mortality, and five (7%) went for discharge against medical advice (DAMA). While doing follow-ups of 64 patients, four (6.5%) rebled, 51 (79.5%) did not report rebleed until one month of follow-up, and nine (14%) did not report to the hospital for any follow-up. Table 3 shows the distribution of patient outcomes according to various factors.

**Table 3:** Distribution of patient outcomes according to various risk factors DAMA: discharge against medical advice, HCV: hepatitis C virus, HBV: hepatitis B virus, NASH: non-alcoholic steatohepatitis, HE: hepatic encephalopathy, ARF: acute renal failure, SBP: subacute bacterial peritonitis, PRBC: packed red blood cell, Hb: hemoglobin, INR: international normalized ratio

	Number		Outcome						Chi-square value	p-value
			DAMA		Discharged		Inhospital or 30-day mortality			
Age group										
31-40 years	8	10.6%	0	0%	8	100%	0	0%	9.6	0.01
41-50 years	20	26%	4	20%	15	75%	1	5%		
51-60 years	25	33.3%	1	4%	23	92%	1	4%		
61-70 years	19	25%	0	0%	16	84%	3	16%		
>70 years	3	4%	0	0%	2	66.7%	1	33.3%		
Sex										
Female	13	17.3%	1	7.6%	11	84.8%	1	7.6%	0.469	0.791
Male	62	82.7%	4	6.4%	53	82.5%	5	11.1%		
Chief complaints										
Hematemesis	24	32%	2	8.6%	19	82.6%	2	8.8%	3.61	0.722
Hematemesis, melena	31	41.3%	3	9.6%	26	83.8%	2	6.6%		
Melena	20	26.7%	0	0%	18	90%	2	10%		

Risk factors										
Alcohol intake	40	53.3%	3	7.5%	34	85%	3	7.5%	0.560	0.967
Drug intake	15	20%	1	6.5%	13	87%	1	6.5%		
HCV	6	8%	0	0%	5	85.4%	1	16.6%		
HBV	2	2.7%	1	50%	1	50%	0	0%		
NASH	6	8%	0	0%	5	89%	1	11%		
No risk factor	6	8%	0	0%	6	100%	0	0%		

Organ dysfunction										
HE	15	20%	1	6.6%	9	60.4%	5	33%	9.654	0.01
Shock	11	15%	3	15.5%	2	52.5%	6	54%		
Respiratory failure	8	11%	1	12.5%	1	12.5%	6	75%		
ARF	11	15%	1	9%	4	36%	6	54%		
SBP	12	16%	2	16.6%	7	58%	3	25%		
Hb (g/dL)										
<5	1	1.3%	0	0%	0	0%	1	100%	29.452	0.001
5-7	32	42.6%	3	9%	26	82%	3	9%		
7-9	37	49.4%	1	2.2%	34	92.4%	2	5.4%		
>9	5	6.7%	1	20%	4	80%	0	0%		
INR										
1-1.5	17	22.7%	3	18%	12	76%	1	6%	8.28	0.03
1.6-2	41	54.7%	0	0%	40	97.5%	1	2.5%		
2.1-2.5	8	10.6%	1	12.5%	5	62.5%	2	25%		
≥2.6	9	12%	1	11.1%	6	66.6%	2	22.2%		
Platelet count										
<50,000	6	8%	1	16.7%	2	33.3%	3	50%	8.524	0.014
50,000-1 lac	49	65.3%	3	6.6%	45	91.8%	1	2.2%		
1-1.5 lac	6	8%	0	0%	5	83.3%	1	16.7%		
≥1.5 lac	14	18.7%	1	7.1%	12	85.8%	1	7.1%		
Rebleed										
No	63	84%	3	5%	58	92%	2	3%	13.427	0.001
Yes	12	16%	2	16%	4	34%	6	50%		
Endoscopic findings										
Variceal bleed	49	65.3%	3	6.5%	41	83%	5	10.5%	0.966	0.616
Non-variceal bleed	26	34.7%	2	7.4%	23	88.9%	1	3.7%		
Timing of endoscopy from admission										
≤6 hours	12	16%	0	0%	12	100%	0	0%	22.289	0.001
6-12 hours	32	42.6%	1	3.2%	30	93.7%	1	3.2%		
12-24 hours	21	27.9%	2	10.5%	18	84.3%	1	5.2%		
>24 hours	10	13.5%	2	20%	4	40%	4	40%		
Rockall score in non-variceal bleed (N=26)										
<3	8	30.8%	2	25%	6	75%	0	0%	14.403	0.006
3-7	16	61.5%	1	6.5%	15	93.5%	0	0%		
≥8	2	7.7%	0	0%	1	50%	1	50%		
PRBC transfusion										
Yes	49	65.3%	4	8.4%	40	79%	5	12.6%	2.567	0.671
No	26	34.7%	1	3.8%	25	92.6%	1	3.8%		

## Discussion

UGIB is a common emergency seen by physicians with significant morbidity and mortality. The first line of investigation for the evaluation of UGIB is endoscopy. Early endoscopy helps in the diagnosis of certain lesions, guiding further care and thereby reducing rebleeding, the requirement for the need for surgery, costs, and the duration of hospitalization.

In the present study, the mean age was 52.19±6.65 years, which is similar to the study done by Kaliamurthy et al. [9], who reported a slightly higher mean age of 55 years. In our study, male predominance (82.7%) was reported, which is similar to the findings of Anand et al. [10], who also reported male preponderance (83.3%). As per the National Family Health Survey, India-5 (NFHS-5), in Punjab, alcohol consumption in the male population is 22.8% as compared to 0.3% in the female population, which seems to be a predominant factor accounting for male predominance in the present study.

Of the patients, 41.7% had both hematemesis and melena. These results are comparable to the study of Anand et al. [10], in which 59.64% presented with both hematemesis and melena.

Chronic alcohol intake was found in 53.3% of patients, which is in concordance with the study results of Mahajan and Chandail [11], where it was 67.09%. This study reported a higher number of diabetics (21.3%) and hypertensives (16%), as compared to studies done by Perveen et al. [2], where diabetics were 6.1% and 6.1% were hypertensives.

Low mean Hb value was observed in melena patients (5.6±1.6 g/dL) as compared to hematemesis patients (7.5±2.9 g/dL), which could be best explained by delayed presentation after bleeding in patients having melena. We found a significant increase in mortality with low Hb levels (p<0.05). This result was in concordance with that of Jain et al. [12]. The above results indicated that significant hemorrhage is associated with increased mortality in study participants.

In the present study, 81.3% had less than 150 × 10^9^ platelets/L, and INR was increased in 77%. In variceal bleeding patients, the platelet count was significantly lower and INR was significantly higher (p<0.05). These results are in concordance with the results of Jain et al. [12], where 77% had low platelet count and INR was increased in 40%. Most of the study population was having chronic liver disease, which could explain low platelet count due to hypersplenism and high PT-INR due to decreased synthetic function of the liver.

We found hepatic encephalopathy as the most common complication, which could be better explained by the high prevalence of chronic liver disease. Contrary to our finding, Prasad et al. [13] observed that shock was the most common complication.

The endoscopic findings of the present study were comparable to studies done by Jain et al. [12], who observed a higher incidence of varices over peptic ulcers. These findings could be due to a higher prevalence of chronic alcohol consumption and chronic viral hepatitis leading to chronic liver disease.

In the present study, while comparing mortality according to the timing of endoscopy, it was observed that delay in endoscopy from admission was associated with an increase in mortality rate. In patients where endoscopy was done within less than 24 hours of admission, an 8.4% mortality rate was observed as compared to 40% in patients where endoscopy was done after 24 hours of admission. Actively bleeding ulcers and vessels were better visualized in early endoscopy and managed with endoscopic hemostatic treatment, resulting in a significant reduction in mortality. The results were similar to those of Cho et al. [14], who showed lower mortality in patients undergoing urgent endoscopy. However, Lim et al. [15] demonstrated that endoscopy within 13 hours of presentation was associated with a lower mortality rate in high-risk, but not low-risk, non-variceal UGIB. However, we had not observed a relationship between the timing of endoscopy and mortality separately in patients with variceal and non-variceal bleeding.

Of the patients, 16% had rebleeding, which was in concordance with the study results of Mahajan and Chandail [11], who reported a 20%-30% rebleeding rate. We reported 50% mortality in rebleeders, and most of the rebleeders have varices. Coronary artery disease (CAD), chronic liver disease, large varices, those undergoing endotherapy, renal failure, low Hb, high pulse rate, low systolic blood pressure, increased INR, those in need of blood transfusion, and Rockall score of >3 were significant predictive factors in rebleeders as compared to non-rebleeders. Booker et al. [16] also reported that renal failure, low Hb, high pulse rate, low systolic blood pressure, prolonged PT, and elevated serum creatinine were strong predictors of rebleed.

Being a specialized referral center in North India, our hospital experiences an influx of critically ill patients, and therefore, our study reported a higher mortality rate as compared to other studies. Financial constraints in a private hospital explained the DAMA rate.

Outcome/mortality

The present study showed that an increase in age was significantly associated with a high mortality rate (p=0.01). Similar results were observed by Perveen et al. [2], who observed higher mortality in the older age group of more than 60 years. The majority of elderly patients had associated comorbidities, which played a significant role in the high mortality rate, and elderly patients have poor tolerance to blood loss due to limited physiological reserve [17].

In our study, 11 patients presented with shock, i.e., low systolic blood pressure (<100 mmHg), and six of them died. A mortality rate of 54% was seen in patients of shock with UGIB. This indicates that shock at the time of presentation is a poor prognostic factor. These results were in concordance with those of the study by Rockall et al. [5], who described shock as a significant independent risk factor for mortality. The left ventricular volume gets compromised in shock, and UGIB can worsen the condition [18].

In the present study, a 54% mortality rate was observed in patients with acute renal failure (ARF), and ARF was significantly associated with mortality (p<0.05). Chaikitamnuaychok and Patumanond [19] also concluded that the severity of UGIB increases with impaired renal function.

Low Hb at the time of presentation was significantly associated with poor clinical outcomes (p=0.001). These results were in concordance with the study results of Chaikitamnuaychok and Patumanond [19], who observed increased mortality with lower presenting Hb levels. When followed over time, the Hb level is a useful indicator of the severity of bleeding.

Most of the patients in the study population have chronic liver disease, which explained the higher number of patients with coagulopathy, and high INR was significantly strongly associated with mortality (p<0.05). Cook et al. [20] also observed that coagulopathy increased the risk of rebleeding and mortality (odds ratio: 4.3, p≤0.001).

Predictors of poor outcome

From the results of the present study, we evaluated age > 60 years, shock, renal failure, ARDS, low Hb, low platelet count, higher PT, variceal bleed, rebleed, late endoscopy, and Rockall score > 8, and all were associated with increased rates of mortality and poor outcome.

There are certain limitations in our study. Our study is a single-center study, we have a relatively smaller sample size, and we had not observed the relationship between the timing of endoscopy and mortality separately in patients with variceal and non-variceal bleeding. Future multicenter studies involving various patient cohorts are warranted.

## Conclusions

Portal hypertension is the most common cause of UGIB, and the most common endoscopic lesions are esophageal varices. Age > 60 years, shock, respiratory failure, low hemoglobin, low platelet count, deranged PT-INR, variceal bleed, renal failure, rebleed, Rockall score ≥ 8 (non-variceal), and late endoscopy are poor outcome predictors. Early and appropriate fluid and pharmacological management and endoscopic hemostatic treatment definitely help reduce morbidity and mortality in patients with UGIB.
